# Would You Believe? A Virus Changes Diabetes Care

**DOI:** 10.1177/1932296820929381

**Published:** 2020-05-27

**Authors:** Cornelis (Cees) J. Tack

**Affiliations:** 1 Radboudumc, Department of Internal Medicine, Nijmegen, The Netherlands

**Keywords:** COVID-19, diabetes, obesity, ICU, remote diabetes care, telehealth

I have learned that a virus can induce unimaginable changes in diabetes care that we have not been able to implement for many, many years. Some information on the ground: the moment I am writing this contribution (April 16, 2020), our University hospital, located in the (south)east of the Netherlands, is over the worst part of the corona virus disease (COVID-19) outbreak. The epicenter of the epidemic in our country was close by and probably related to the tradition in the south to celebrate carnival in large crowds amid February—with part of them probably being infected during winter holidays in Austria and northern Italy. As the peak was expected to come, the hospital was completely reorganized into COVID-19 wards, COVID-19 suspected wards, and non-COVID wards and clinics; the ICU capacity was doubled; all nonurgent care and procedures were discontinued; and fourfold staffing on call rotations. While none of us had ever witnessed a similar situation, these huge organizational changes went in fact relatively smooth and we have always been in control. Currently the number of new cases has clearly decreased, some COVID wards are closed down, and regular care will resume, although we believe that “COVID-care” will remain part of our workflow for quite a while.

A first intriguing observation during the peak of the outbreak was that the number of non-COVID emergencies, for example, myocardial infarction, stroke, severe trauma, and new cancer cases dramatically decreased. While this may be partly explained by delayed demand for care, it must partly be real and due to the lockdown situation. And we haven’t seen a single case of diabetic keto-acidosis (DKA) during this period—most cases admitted for DKA are patients known with diabetes but with poor self-care. Apparently, the COVID crisis strongly stimulates self-care (see below).

The relation between COVID-19 and diabetes is well described; clearly patients with diabetes are vulnerable to develop more serious infections with poor outcomes, a fact that is every day in all media and now known to everyone. Less well described is the fact that most patients admitted to the ICU for COVID-19-related Adult Respiratory Distress Syndrome (ARDS) are obese, not necessarily in the context of diabetes, mostly males with abdominal obesity and females on the ICU reportedly also having apple-shaped obesity. Some earlier series with ARDS due to SARS and MERS have reported similar observations. Again this fact has been broadly covered in the media, also generating “fat-shaming” and suggestions to deprive fat people from ICU admission. The result is that patients with diabetes but also obesity now seem clearly aware of their risk and most are scared.

Being a tertiary diabetes center we serve complicated patients, most on insulin therapy, the vast majority nowadays using flash glucose monitoring, ≈40% of the type 1 population using a pump, a few on closed-loop systems. Extensive use of continuous glucose monitoring systems, an advanced electronic health care record (EPIC) allowing direct patient access into their data and direct communication with their health care providers, the already available option for secured video consulting, together with a rather high education level of the population, high Wi-Fi density, and use of digital tools in the country seemed to be ideal ingredients to move to remote diabetes care and decrease the number of clinic visits. This has been an ongoing project for years, with little progress. Many financial, organizational, technical, and physician-related barriers were identified, also from patients: few people would upload their data ahead of a visit and many people expressed their concern that remote care simply meant less care and cost-saving—even the tech-savvy often preferred an in-person clinic visit.

But this virus has changed it all. With all clinic visits now altered into telephone or video consults, most barriers have suddenly disappeared; reimbursement has been arranged, technically it does work and the number of patients uploading data has doubled; patients now do realize that diabetes and obesity is a problem and seem motived to deal with it (success remains to be seen). Many now agree that remote care is probably as good as traditional care and a lot more convenient. In the future I predict that these changes will sustain. For us as diabetes care providers now the task is to grasp this momentum—in terms of Churchill—to “Never waste a good crisis.”

